# Study on Horizon Scanning with a Focus on the Development of AI-Based Medical Products: Citation Network Analysis

**DOI:** 10.1007/s43441-021-00355-z

**Published:** 2021-11-22

**Authors:** Takuya Takata, Hajime Sasaki, Hiroko Yamano, Masashi Honma, Mayumi Shikano

**Affiliations:** 1 Faculty of Pharmaceutical Sciences, Tokyo University of Science, Tokyo, Japan; 2 Institute for Future Initiatives, The University of Tokyo, Tokyo, Japan; 3 Department of Pharmacy, The University of Tokyo Hospital, Tokyo, Japan

**Keywords:** Horizon scanning, Citation network, Delivery of health care/trends, Diagnostic imaging, Artificial intelligence

## Abstract

Horizon scanning for innovative technologies that might be applied to medical products and requires new assessment approaches to prepare regulators, allowing earlier access to the product for patients and an improved benefit/risk ratio. The purpose of this study is to confirm that citation network analysis and text mining for bibliographic information analysis can be used for horizon scanning of the rapidly developing field of AI-based medical technologies and extract the latest research trend information from the field. We classified 119,553 publications obtained from SCI constructed with the keywords “conventional,” “machine-learning,” or “deep-learning" and grouped them into 36 clusters, which demonstrated the academic landscape of AI applications. We also confirmed that one or two close clusters included the key articles on AI-based medical image analysis, suggesting that clusters specific to the technology were appropriately formed. Significant research progress could be detected as a quick increase in constituent papers and the number of citations of hub papers in the cluster. Then we tracked recent research trends by re-analyzing “young” clusters based on the average publication year of the constituent papers of each cluster. The latest topics in AI-based medical technologies include electrocardiograms and electroencephalograms (ECG/EEG), human activity recognition, natural language processing of clinical records, and drug discovery. We could detect rapid increase in research activity of AI-based ECG/EEG a few years prior to the issuance of the draft guidance by US-FDA. Our study showed that a citation network analysis and text mining of scientific papers can be a useful objective tool for horizon scanning of rapidly developing AI-based medical technologies.

**Supplementary Information:**

The online version contains supplementary material available at 10.1007/s43441-021-00355-z.

## Introduction

The application of innovative technologies to the development of medical products is expected as a potential new treatment or diagnostic tool for various diseases. Conversely, in some cases, the application of conventional development and evaluation concepts and/or regulatory frameworks to innovative technologies is inappropriate. In some cases in the past, a guidance document was issued when the clinical development is about to begin, the development of companion diagnostics in Japan, the U.S., and the EU. However, the guidance must be provided earlier, such as before starting clinical development planning. Therefore, the early identification of innovative technologies with a potential application to medical products through horizon scanning would encourage regulatory authorities to establish new approaches to assess their quality, efficacy, and safety to advice developers and revise their regulations if necessary. Doing so contributes to timely patient access and improve the benefit/risk ratio of the product [1].

The International Coalition of Medicines Regulatory Authorities (ICMRA), consisting of regulatory authorities, has recognized the need to respond quickly to innovative technologies and promotes the use of “horizon scanning” to identify such technologies [2]. The ICMRA Innovation concept note [3] describes horizon scanning as a broad-reaching information-gathering monitoring activity to anticipate emerging products and technologies and potentially disruptive research avenues. Two major methods exist for acquiring the data needed to create high-quality horizon scanning [4]. The expert-based approach mainly uses the tacit knowledge of domain experts, such as the Delphi method. Traditionally, horizon scanning has been conducted predominantly in Europe for policy making, scientific research funding, and health-care budgeting purposes, by surveying a variety of sources such as the Internet, government, international organizations and companies, databases, and journals [5, 6]. This type of expert-based approach is very difficult to implement in the current information explosion. Moreover, individual experts must subdivide their domain of expertise to keep up with the growth of their respective domains, which makes their perception of the big picture extremely subjective [7]. Computer-based approaches collect and analyze vast amounts of formal knowledge, such as articles, patents, and newspapers. Recently, the European Commission (EC) published reports, such as “Weak signals in Science and Technologies 2019 Report” based on Tools for Innovation Monitoring (TIM) [8] that use text mining and keywords in the scientific literature. The Japanese National Institute of Science and Technology Policy (NISTEP) also uses a digital tool to analyze academic papers; the top 1% of citations contributes to science and innovation policy planning.

These cover the medical field as a sub-survey of the overall science survey and are used in efforts to identify and evaluate advanced technologies.

Hines et al. reported that, in the medical and health-care field, most horizon-scanning methods used manual or semi-automated, with relatively few automated aspects, which may be resolved in the not-too-distant future via the rapidly evolving fields of machine learning and artificial intelligence [6]. To solve this challenge, a computer-based approach can complement the expert-based approach as it fits the scale of the information [9, 10] because they are compatible with the scale of the information. The two types of computer-based approaches are citation mining and text mining.

The citation-based approach assumes that the cited papers and their research topics are similar. Analyzing this citation network allows us to understand the structure of the research areas constituting the large volume of papers that we can read. These methods have been widely used as powerful tools to visualize and understand the structure of a research field and to identify new trends and research directions; they also have been proven effective in various studies [11–13]. For example, Kajikawa et al. [7] used citation network analysis to track emerging research areas in the field of sustainable science effectively and efficiently. Many fields have applied similar approaches, including energy research [14], regenerative medicine [15], robotics, and gerontology [16]. Sakata et al. [17] proposed a meta-structure of academic knowledge on patent and innovation research to effectively assist policy discussions on intellectual property system reform. They have shown that network analysis and machine learning methods are useful for understanding and predicting the development of technologies such as solar cells [18] and nanocarbons [19].

Many fields have used also text mining to analyze technology trends; Kostoff et.al. (2004) analyzed multi-word phrase frequencies and phrase proximity to extract energy-related taxonomic structures [20]. Another study discussed the trend in the field of information security by creating a network of co-occurring words and focusing on clusters with network centralities [21]. Ohniwa et al. (2010) focused on the MeSH terms included in the top 5% of the increase rate in a given year in the field of life science [22]. A study to discuss a community’s the future prospects by calculating the cosine similarity of terms in the session content from the data of conference proceedings focused on the field related to the World Wide Web [23].

R&D strategists and policymakers in many fields find citation network analysis and text mining useful to understand the broad scope of scientific and technological research.

It is difficult to understand the semantics of clusters based on citation relations alone. Text mining can reveal subject relationships across citations and provide insights into the diffusion of knowledge into interdisciplinary research and development. The addition of text mining to citation-based bibliometrics makes accessible the large-scale multigenerational citation studies necessary to display the full impact of research [24].

Text mining is extremely sensitive to certain terms. When only text mining is used, the problem of terminological distortions cannot be ignored. In addition, it is difficult to separate homonyms that are used in different fields with different meanings. Hao et al. (2018) attempted to identify research fronts using only text mining in the medical field [25]. They highlighted the challenges of clustering by text similarity, which makes the results vulnerable to the method selection. At the same time, they observed that citation relationships are highly valuable in explaining relationships in scientific knowledge.

Therefore, there are challenges in analyzing trends using only one citation network and text mining. The associations between papers in citation networks reflect authors' background knowledge which cannot be extracted by simple text mining.

Our study proposes an objective methodology for horizon scanning that identifies innovative technologies to be applied to medical products from entire research papers in the target field using citation network analysis methods and text mining. The three types of citation network analysis are direct citation, bibliographic merging, and co-citation. Existing studies have shown that direct citation is the most appropriate for obtaining leading-edge information on trends [26]. Other fields have widely used the approach of clustering the subject area into subcategories by direct citation networks and interpreting the contents of the clusters by text mining [7, 14–17], but insufficient examples exist of the application of advanced technologies in medical-related fields. We focus on AI-based medical image analysis as a retrospective example of AI-based medical devices that have been developed in recent years, applied in many fields, and selected for consideration in ICMRA [1].

## Methods

### Extraction of Paper Data for Analysis

We used “convolutional” OR “deep learning” in the review article of medical image analysis [27]; we used “machine-learning” to include a wide range of conventional studies. As a result, we obtained 140,794 papers that contain “convolutional*” OR “machine-learning” OR “deep-learning” from the SCI (Science Citation Index) and SSCI (Social Sciences Citation Index) indexed by Web of Science Core Collection (WoS, Clarivate analytics), between January 1, 1900, and December 31, 2020, (1900–2020). This database has the longest history of containing bibliographic information from academic papers. It is also used for many bibliometric analyses because of its excellent searchability and comprehensiveness as a database platform [7, 14–17]. In addition to the data in 1900–2020, we created datasets for 1900–2012, 1900–2013, 1900–2014, 1900–2015, 1900–2016, 1900–2017, 1900–2018, and 1900–2019 and identified the cluster that contains key articles for each year.

To track the development history of AI-based medical image analysis and to select keywords for the extraction of the papers for citation network analysis, we selected 13 key articles [28–39] (Table 1 presents eight articles included in the analysis data), including several papers cited in the review article [28] on the application of deep learning in medical image analysis and a study [39] that led to the clinical development of IDx-DR, a retinal imaging software approved as a medical device by the US Food and Drug Administration (FDA) in 2018.

**Table 1:** Key articles and the clusters in which they are contained

Label	Paper title	published year	Web of science	
			Cluster No	Times cited within each cluster
A	Gradient-based learning applied to document recognition. [27]	1998	1	1590
B	Learning hierarchical features for scene labeling. [29]	2012	1	304
C	Imagenet classification with deep convolutional neural networks. [30]	2012	1	1742
D	Deep learning. [34]	2015	3	1825
E	Pulmonary nodule detection in CT images: false positive reduction using multi-view convolutional networks. [36]	2016	3	239
F	Improved automated detection of diabetic retinopathy on a publicly available dataset through integration of deep learning. [37]	2016	3	151
G	Dermatologist-level classification of skin cancer with deep neural networks. [38]	2017	3	1015
H	A survey on deep learning in medical image analysis. [28]	2017	3	1127

The key articles that have contributed to the development of AI-based medical image analysis were selected based on a review article on AI-based medical image analysis [32]. The clusters obtained from the citation network analysis of these articles are indicated. The clusters are numbered in descending order of the number of constituent papers included. The cells for papers not included in the analysis were shadowed. 8 articles are listed, excluding the 5 articles [31–33, 35, 39] that were excluded

### Citation Network Analysis

**Fig. 1:**
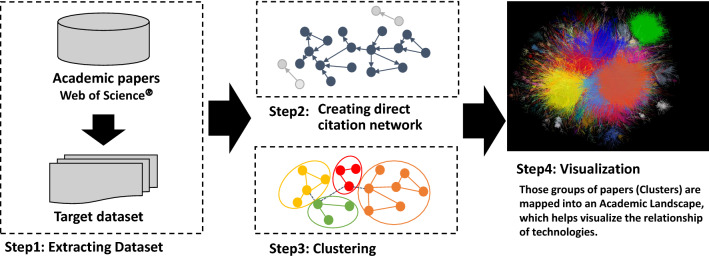
Steps of clustering and making Academic Landscape based on citation network. This figure has been published in reference [10]. The procedure of the citation network is as follows: (1) Extract the dataset of academic papers for analysis. (2) To extract the data, convert the citation network into an unweighted network with papers as nodes and citation relationships as links. (3) Divide the network into several clusters by using the topological clustering method. (4) Use a large graph layout (LGL), based on a force-direct layout algorithm, to display the largest connected component of the network to generate coordinates for the nodes in two dimensions and to visualize the citation network by expressing inter-cluster links with the same color.

In this study, we converted the citation network into an unweighted network with papers as nodes and citation relationships as links. Papers with no citations as the largest component were considered digressional and were ignored in this study (Step 2 in Fig. 1). The core paper with the highest number of citations appears at the center of the citation relations. Papers with no citation relationships with other papers were considered deviant and ignored in this study. The network was then divided into several clusters using the topological clustering method. Topological clustering is a clustering method based on the graph structure of a network; here, we use modularity maximization. A cluster module in a citation network is a group of papers in which the citation relations are divided by using a modularity (*Q* value) maximization method and are densely aggregated (Louvain method) [19, 40]. The modularity maximization method appreciates network partitioning so that the intracluster is dense and the inter-cluster is sparse. The modularity maximization method determines an optimal partitioning pattern by extracting the partitioning pattern that maximizes the modularity using a greedy algorithm. *Q* is an evaluation function of the degree of coupling within a cluster and between clusters, as follows:

$$Q=\frac{1}{2m}\sum_{i,j}\left({A}_{ij}-\frac{{k}_{i}{k}_{j}}{2m}\right)\delta \left({c}_{i},{c}_{j}\right),$$

where ${A}_{ij}$ represents the weight of the edge between $i$ and $j$, ${k}_{i}={\sum }_{j}{A}_{ij}$ is the sum of the weights of the edges attached to vertex i, c_i_ is the community to which vertex *i* is assigned, δ-function δ(*u*, *v*) is 1 if $u=v$ and 0 otherwise, and $m=\frac{1}{2}{\sum }_{ij}{A}_{ij}$.

The clusters are assigned labels corresponding to the size of the number of papers included. The characteristics of each cluster were confirmed by extracting a summary of frequently cited academic papers in the cluster and the characteristic keywords in the cluster.

Moreover, we computed the term frequency-inverse cluster frequency (TF-ICF) to extract the characteristic keywords of each cluster. The TF gives a measure of the importance of a term in a particular sentence, whereas the ICF provides a measure of the general importance of a term. The TF-ICF of a given term *i* in a given cluster *j* is given by

$$\text{TF-ICF}={tf}_{i,j}\cdot {icf}_{i}={tf}_{i,j}\cdot \log (N/{cf}_{i}),$$

where *N* is the total number of sentences. Each cluster was labeled based on the resulting keywords and sentences.

To confirm the trends in the research field, we extracted the mean or median year of publication of papers in each cluster, as well as information on journals, authors, and affiliated institutions.

After clustering the network, visualization is converted to intuitively infer relationships among these clusters. We used a large graph layout (LGL) based on a force-direct layout algorithm [41, 42]. This layout can display the largest connected component of the network to generate coordinates for nodes in two dimensions. We visualize the citation network by expressing inter-cluster links with the same color (Step 4 in Fig. 1). However, the position of the clusters and the distance between clusters did not indicate an approximation of the content. Figure 1 shows an overview of this process.

For the extracted dataset, we converted the citation network into an unweighted network with papers as nodes and citation relationships as links (Step 2). The network was then divided into several clusters using the topological clustering method (Step 3). Moreover, a LGL, based on a force-direct layout algorithm, displayed the largest connected component of the network to generate coordinates for the nodes in two dimensions, visualizing the citation network by expressing inter-cluster links with the same color (Step 4).

## Results

### Results of Citation Network Analysis

**Fig. 2:**
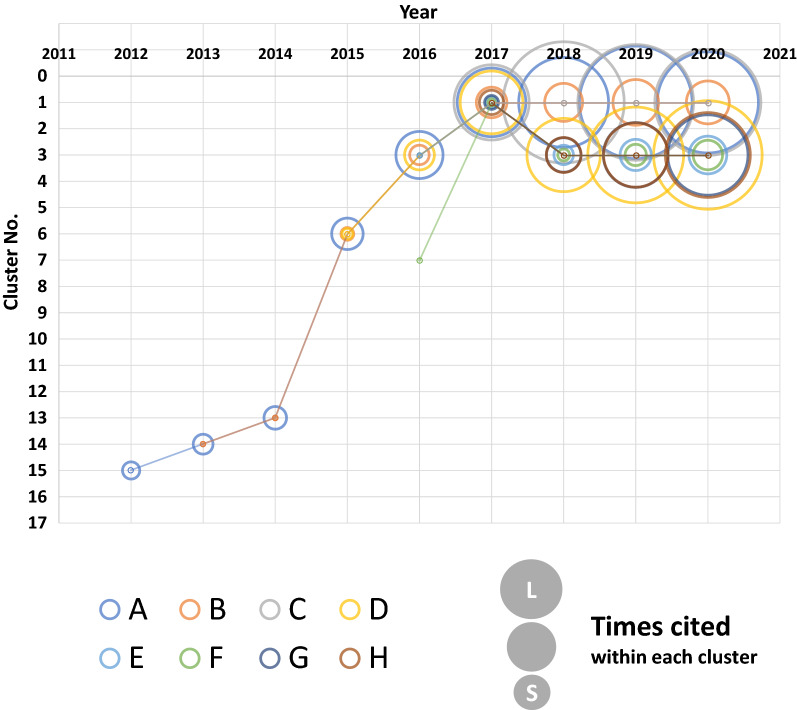
Tracking clusters containing key articles. We analyzed papers obtained from WoS published up to the indicated years. We plotted the cluster numbers that contained the eight key articles shown in Table 1, with the circle sizes representing the approximate number of citations in the cluster for each paper

We analyzed 140,794 papers and found that 119,553 (85%) formed a citation network. We divided this network into 36 clusters by extracting the largest linkage component from all linkage components via direct citation of papers (excluding the gray linkage not involved in cluster formation shown in Figs. 1, 2). The contents of the top 10 clusters, which contain approximately 75% of the papers in a citation network, were estimated from the characteristic keywords appearing in each cluster and the titles and abstracts of the papers with the highest number of citations. The cluster numbers (number of papers) and their contents are as follows:

**Cluster 1** (14,033): Basic studies on deep learning and convolutional neural networks (CNNs), including geographic information system (GIS) image analysis using remote sensing.

**Cluster 2** (13,309): Drug discovery technologies related to proteins, peptides, *etc.*, using machine learning.

**Cluster 3** (10,992): Applied research in medical image analysis.

**Cluster 4** (9867): Feature classification using ensemble methods to increase accuracy by combination.

**Cluster 5** (7829): Natural language processing of clinical records.

**Cluster 6** (7412): Application of deep learning to fault diagnosis, for example, motor condition monitoring for machines running on electric motors.

**Cluster 7** (6571): Machine learning (ML) and data mining (DM) methods for cyber analysis.

**Cluster 8** (5815): Application to traffic flow information analysis for the implementation of intelligent transport systems.

**Cluster 9** (4371): Single-image super-resolution (SR) to reconstruct high-quality data.

**Cluster 10** (4333): Classification of individuals based on the analysis of text information from social media, such as emotions and behavior.

Table 1 presents the clusters in which key articles were included. Three papers (labeled A, B, and C) based on image recognition were found in clusters 1 and 5 (labeled D, E, F, G, and H) on image diagnosis in cluster 3, including the review article “Deep Learning” [34] (labeled D), which is often cited in medical field papers. This indicates that we appropriately formed clusters related to medical imaging in cluster 3.

### Tracking the Time Series of Key Articles

We analyzed papers published each year and identified the cluster containing the key papers in Table 1 and the number of citations within the cluster to assess the position of the research on medical imaging in the past. As shown in Fig. 2, all the papers were included in the same cluster until 2015 and the rank of cluster number increased by one until 2014. In 2015, the number of papers in this field increased rapidly and the rank of cluster numbers rose from 13th in 2014 to 6th, suggesting that great scientific attention has increased. In 2016, a key paper on the imaging diagnosis of diabetic retinopathy (F in Table 1) was in cluster 7, which comprised papers on medical image analysis, and the other seven key articles were in cluster 3. Subsequently, in 2017, cluster 1 contained all the key articles, but from 2018 onward, a new separate cluster containing papers on image analysis using deep learning was formed. It should be noted that the number of citations of key articles also increased.

Thus, most key articles were in one or two clusters, suggesting that we properly formed the clusters related to the targeted AI-based medical image analysis. The research status of the clusters can also be confirmed by the cluster numbers, which reflect the number of papers comprising the cluster and the number of citations of the key articles.

### Recent Research Trends in AI-Based Medical Products

**Fig. 3:**
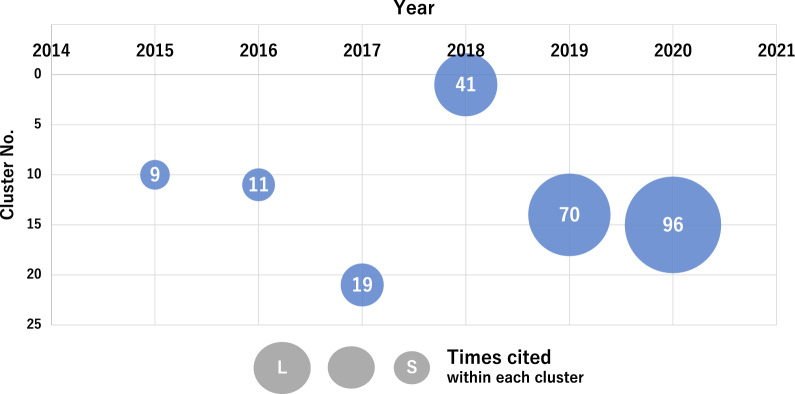
Tracking clusters related to ECG and EEG. We analyzed papers obtained from WoS published up to the indicated years. A cluster number indicates the cluster on ECG and EEG. The circle sizes indicate the approximate citation frequency of the key article, [73] and the number in each circle represents the number of citations in the cluster. Clusters on ECG and EGG were first detected in 2015 as cluster number 10 and were classified into cluster numbers 11, 21, 1, 15, and 15 for 2016, 2017, 2018, 2019, and 2020, respectively

To detect the latest research trends in AI-based medical products, we focused on “younger” clusters with an average publication year later than 2017 as research progress could be observed over three years for AI-based medical image analysis (Fig. 3). We re-analyzed clusters 3, 15, 12, 5, 13, and 2, which we considered to be closely related to AI-based medical technologies. We listed these clusters in order of average publication year. Table 2 lists the sub-clusters formed by re-analysis of the most cited articles (hub-paper) [34, 43–74] in each subcluster, suggesting recent research trends in this field as follows:

**Table 2:** Sub-clustering results for clusters of AI-based medical technologies

Cluster name	Average year	The number of papers	Top keywords	Hub papers
Cluster3	2018.8	10,992	Segmentation, cancer, radiomics	Deep learning [34]
Sub3-1	2018.3	1179	Glaucoma, optical coherence tomography, retinal	Development and Validation of a Deep Learning Algorithm for Detection of Diabetic Retinopathy in Retinal Fundus Photographs [43]
Sub3-2	2019.1	1017	Brain tumour segmentation, MRI, lesion	Efficient multi-scale 3D CNN with fully connected CRF for accurate brain lesion segmentation [44]
Sub3-3	2018.9	1011	Whole slide, cancer, pathology	Diagnostic Assessment of Deep Learning Algorithms for Detection of Lymph Node Metastases in Women With Breast Cancer [45]
Sub3-4	2019.1	1004	Radiograph, bone age, aneurysm	Deep Learning at Chest Radiography: Automated Classification of Pulmonary Tuberculosis by Using Convolutional Neural Networks [46]
Sub3-5	2018.8	980	Radiomics, glioma, MRI	Machine Learning methods for Quantitative Radiomic Biomarkers [47]
Cluster15	2018.3	3202	EEG (electroencephalogram), ECG (electrocardiogram), seizure	Real-Time Patient-Specific ECG Classification by 1-D Convolutional Neural Networks [48]
Sub15-1	2019.0	606	ECG, arrhythmia, heartbeat	Real-Time Patient-Specific ECG Classification by 1-D Convolutional Neural Networks [48]
Sub15-2	2017.4	560	EEG, brain–computer interface, motor imagery	A novel deep learning approach for classification of EEG motor imagery signals [49]
Sub15-3	2018.5	394	Seizure, EEG, epilepsy	Deep convolutional neural network for the automated detection and diagnosis of seizure using EEG signals [50]
Sub15-4	2018.3	379	Emotion, EEG, physiological signal	EEG-Based Emotion Recognition in Music Listening [51]
Sub15-5	2018.3	239	Surface electromyography, myoelectric, prosthesis	Electromyography data for non-invasive naturally-controlled robotic hand prostheses [52]
Cluster12	2018.3	4101	Gait, activity recognition, video	3D Convolutional Neural Networks for Human Action Recognition [53]
Sub12-1	2018.8	694	Action recognition, video, convolutional neural network	3D Convolutional Neural Networks for Human Action Recognition [53]
Sub12-2	2018.6	530	Human activity recognition, wearable sensor, accelerometer	Deep Convolutional and LSTM Recurrent Neural Networks for Multimodal Wearable Activity Recognition [54]
Sub12-3	2018.2	463	Facial expression, emotion, ck +	Facial expression recognition based on Local Binary Patterns: A comprehensive study [55]
Sub12-4	2017.4	379	Gait, parkinson, walking	A machine learning approach for automated recognition of movement patterns using basic, kinetic and kinematic gait data [56]
Sub12-5	2018.8	251	Hand pose, sign language, human pose	Real-Time Continuous Pose Recovery of Human Hands Using Convolutional Networks [57]
Cluster5	2017.4	7829	Clinical text, disease, electronic health record	Predicting the Future—Big Data, Machine Learning, and Clinical Medicine [58]
Sub5-1	2016.7	828	Clinical text, radiology report, electronic health record	2010 i2b2/VA challenge on concepts, assertions, and relations in clinical text [59]
Sub5-2	2018.3	759	Recidivism, treatment effect, uplift modelling	Machine Learning: An Applied Econometric Approach [60]
Sub5-3	2019.0	753	Readmission, patient, electronic health record	Scalable and accurate deep learning with electronic health records [61]
Sub5-4	2018.1	731	Sepsis, acute kidney injury, ICU	An Interpretable Machine Learning Model for Accurate Prediction of Sepsis in the ICU [62]
Sub5-5	2018.9	710	Coronary artery, cardiac, angiography	A combined deep-learning and deformable-model approach to fully automatic segmentation of the left ventricle in cardiac MRI [63]
Cluster13	2017.6	3800	Disorder, brain, schizophrenia	Single subject prediction of brain disorders in neuroimaging: Promises and pitfalls [64]
Sub13-1	2017.2	546	Schizophrenia, psychosis, bipolar disorder	Using Support Vector Machine to identify imaging biomarkers of neurological and psychiatric disease: A critical review [65]
Sub13-2	2018.6	441	Alzheimer, MCI (mild cognitive impairment), disease	Hierarchical feature representation and multimodal fusion with deep learning for AD/MCI diagnosis [66]
Sub13-3	2017.1	433	MCI, Alzheimer, mild cognitive impairment, impairment	A review on neuroimaging-based classification studies and associated feature extraction methods for Alzheimer's disease and its prodromal stages [67]
Sub13-4	2018.3	350	Suicide risk, depression, mental health	Predicting Risk of Suicide Attempts Over Time Through Machine Learning [68]
Sub13-5	2018.1	315	Autism spectrum disorder, child, ADHD	Identification of autism spectrum disorder using deep learning and the ABIDE dataset [69]
Cluster2	2016.4	13,309	Protein, drug discovery, peptide	Random forests [70]
Sub2-1	2016.4	2056	Ligand, drug, virtual screening	Deep Neural Nets as a Method for Quantitative Structure–Activity Relationships [71]
Sub2-2	2017.3	1873	Gene, random forest, cancer	Random forests [70]
Sub2-3	2018.0	1546	Enhancer, gene, RNA	Predicting the sequence specificities of DNA- and RNA-binding proteins by deep learning [72]
Sub2-4	2018.6	1158	Ligand, patient, NGC (NIDA Genetics Consortium)	Scikit-learn: Machine Learning in Python [73]
Sub2-5	2016.5	1027	DNA binding protein, peptide, amino acid composition	Predicting protein structural classes for low-similarity sequences by evaluating different features[74]

The clusters of AI-medical technologies were re-analyzed and the characteristics of the top five sub-clusters, that is, the number and average of publications of constituent papers, specific keywords, and the title of hub paper are shown

**Cluster 3** Applied research in medical image analysis.

**Cluster 15** Electrocardiogram, electroencephalogram, and other electrical biosignals of human activity.

**Cluster 12** Human activity recognition.

**Cluster 5** Natural language processing of clinical records. Cluster 13: Neuroimaging analysis.

**Cluster 2** Drug discovery with machine learning related to proteins, peptides, *etc.*

Among these AI-based medical technologies, EEG analysis was identified for applications in epileptic seizure prediction, emotional analysis, and brain–computer interfaces, for which the FDA issued draft guidance on non-clinical and clinical trials in 2019.

Electrocardiograms (ECGs) and electroencephalograms (EEGs) in cluster 15 are most likely to be applied to new medical devices; therefore, we tried to follow the cluster containing a key paper on the application of deep learning to EEG analysis [75], which was one of the triggers for the development of this field. During 2015–2016, the article was included in the same cluster as other neuroimaging techniques, such as MRI (MEG, fNIRS, etc.). In 2017, the key article was found in a separate cluster numbered 20 from other neuroimaging techniques, suggesting that a new cluster specific to the application of deep learning to EEG was formed. Then, in 2018, we included the article in cluster 1 of the applications of deep learning in various fields but was included in specific clusters re-formed, numbered 14 and 15 in 2019 and 2020, respectively; the number of citations of the article increased. This suggests that research in this field has developed rapidly since 2017.

## Discussion

In this study, we examined the possibility of using this analysis method for horizon-scanning targeting AI-based medical image analysis. IDx-DR, an image-analysis software for the automatic diagnosis of diabetic retinopathy, received FDA certification in 2018. The AI characteristics are self-learning, the algorithm for learning data during the development of a medical product is in a black box, and performance changes as the product continues learning during clinical use. This has become an interesting dilemma for regulators [76].

We assessed the feasibility of using citation network analysis and text mining to identify trend history in AI-based medical image analysis research and development as follows: Research on convolutional neural networks (CNNs), the current leading technology in deep learning that arose in the 1970s, renewed interest in neural networks was Werbos's multi-layer networks [77]. LeNet [54], a CNN-based handwritten number recognition system—was developed and succeeded by a CNN called AlexNet [30], which is a key trigger for renewed interest in neural networks. Later, the U-net [33] architecture was proposed, which consists of an upsampling section that uses "up" convolution to increase the image size. Furthermore, the combination of CNNs and recurrent neural networks (RNNs), represented by long short-term memory (LSTM), has been applied to analysis involving time-series data [28, 54].

We evaluated 13 key articles, including these milestones in the development of AI-based medical image analysis, to determine how citation network analysis can capture key articles. We identified eight articles in one or two clusters (Table 1), with a concentration of the characteristic keywords of the clusters, and the titles and abstracts of the articles with the highest number of citations confirmed that the clusters were related to AI-based medical image analysis and that identifying actual research trends was possible. Moreover, we analyzed the papers reported each year and found that the number of constituent papers of the cluster containing the key articles increased dramatically after 2014, with the rank rising from 13 to 6th, suggesting that the technology related to diagnostic imaging has progressed dramatically. This might have led to a major clinical trial of IDx-DR in 2017. Since then, research activity has increased in this field, as can be seen from the rank of cluster numbers and number of citations in the key articles.

We did not include five of the 13 selected articles in the analysis: three papers were not included in the WoS and the other two [31, 39] on clinical evaluation were not found with the set query, because there was no mention of the underlying technology in the abstract or title, and the methods were mainly described as product names or computer detection in either paper.

Next, we explored trends in the development of new medical products using AI by re-analyzing “young” clusters with a late average of the publication year of constituent papers to identify more specific topics by sub-clustering (Table 2). This allowed us to objectively look at the landscape of AI-based medical technology. We focused on EEG and ECG, which have the potential to lead to the development of new medical devices, and followed the cluster containing the key article on this topic. As shown in Fig. 3, the increase in constituent papers and citations of key articles suggested that this topic developed significantly between 2017 and 2018, a couple of years before the FDA issued a guidance draft on brain–computer interfaces in 2019, which was finalized in 2021 [78]. Regarding the FDA’s activity, a public workshop was held on November 21, 2014, to promote open discussion of scientific and clinical considerations related to the development of BCI devices, suggesting that the FDA might consult public on product development. The ECG is already at the stage of realization in smartwatches and other devices and was judged to be of low novelty. The FDA has already approved the app for the Apple Watch®.

This study also showed that analysis every several months might allow us to identify the candidate topics for further investigation through the rapid rise of the rank of cluster number, i.e., a sharp increase in constituent papers (2014–2015 in Fig. 2 and 2017–2018 in Fig. 3), or the emergence of a new cluster spun out of the original one (2017–2018 in Fig. 2 and 2016–2017 in Fig. 3), which may be a signal of significant research progress.

This analysis has the following limitations. Which would be detected by this method as well. Therefore, it is necessary to determine whether the candidate topic is a good idea or We included papers in major journals in WoS relatively quickly after publication, but there might be a delay of approximately six months for almost all journals and some research areas may not be reflected in WoS sufficiently quickly, which may delay the identification of research trends. Until the birth of Scopus and Google Scholar in 2004, WoS was the only tool for citation analysis [79]. Even today, WoS is known to have a longer record period than Scopus and is one of the most effective databases in the field of history. In addition to WoS, Scopus and PubMed have also become powerful databases, and future studies are needed to evaluate the robustness of those databases. Although this paper does not show these data, we also analyzed the papers obtained from PubMed; however, approximately 30% of the papers formed a citation network and only five of the 13 key articles were included. One possible reason for not being able to extract appropriate research papers from PubMed was that many papers did not use terminology related to AI-based technologies. This suggests that the choice of the literature database according to the target technology is also critical. Furthermore, research results in the field of machine learning, which covers basic technologies in the field of AI and other informatics fields, tends to be published as proceedings of international conference or arXiv.com as preprints than peer-reviewed journals, where researchers can directly exchange papers with each other via the Internet; therefore, the latest results cannot be covered by databases of academic papers, such as WoS or PubMed. A comparison of peer-reviewed journal-based analysis and proceeding—or preprints-based analysis—needs to be conducted in the future.

Experts who have a deep understanding of innovative technologies would be able to predict the development of medical products based on the technology. However, it might sometimes be inappropriate to narrow the scope of consideration based solely on experts’ opinions [80]. Extracting a limited number of novel topics that may affect pharmaceutical regulations from a vast amount of information on a human basis is difficult and using a computer-based method (such as this study) is reasonable and appropriate. This study assumes that the ultimate users are regulators who evaluate technologies in the mid to long term. Because policymakers and decision makers are not always experts in their fields, providing the status of the academic field in a systematic method supports decision making that can be reproduced by anyone. In our study, we used citation network analysis and text mining to classify the entire papers in the target field in terms of research topic. Furthermore, we identified the topics of the clusters based on the characteristic cluster keywords and titles of the most cited papers. We objectively evaluated the popularity and novelty of a topic based on the number of papers and the median year of publication. We consider that the feature of the method is suitable for a primary screening by regulators to pick up candidate topics from wide range of scientific fields, and the topics would be further evaluated based on the opinion of experts of the topic and other sources such as patents.

We considered that limiting the search to papers in the clinical development stage was rather inappropriate because the purpose of horizon scanning is to detect technologies that have the potential to reach clinical development in the pre-clinical stage. When searching for papers on clinical development, papers on related technologies in the earlier stages are less likely to not include in the analysis, which involves the risks that do not reflect the overall picture of the field. The overall landscape of R&D can be grasped more objectively by analyzing a wide range of papers, for example and then target cluster, the cluster on clinical development is obtained by clustering and re-clustering. Information on the clinical development stage can be directly and timely obtained from clinical trial registries such as ClinicalTrials.gov. The information provided by these other tools from analysis such as “Tools for Innovation Monitoring (TIM)” is useful for determining the query for the papers data in our method.

Another possible bias, as mentioned [81], is that researchers mainly check and cite papers written in their native language or journals they contribute to, or that they tend to search and cite papers using the same terminology and not others, even when the technological meaning is the same.

Considering the opinions of experts in the field regarding candidate topics to be investigated will help in overcoming the aforementioned limitations. Our method provides information about the median and average year of publication of the papers in the cluster and the newness of the Hub paper, but prioritization requires the perspective of an expert in the field. Academic size and speed of discussion do not necessarily determine prioritization; however, depending on the social demands and feasibility of the technologies included in the individual topics. Hence, a content-based evaluation is necessary.

## Conclusion

This study showed that citation network analysis and text mining for bibliographic information analysis of the rapidly developing field of AI-based medicine can be used for horizon scanning for medical products that require new assessment approaches. We detected recent research developments, including AI-based ECG/EEG. We suggest that this method be used as a primary screening tool for horizon scanning, and that the analysis results be used more effectively and appropriately by incorporating the opinions of experts.

## Supplementary Information

Below is the link to the electronic supplementary material.

Supplementary file1 (DOCX 39 kb)
