# Supplementary material for: Study on Horizon Scanning with a Focus on the Development of AI-Based Medical Products: Citation Network Analysis
Source: Ther Innov Regul Sci. 2021 Nov 22;56(2):263–75. doi: 10.1007/s43441-021-00355-z (PMC8854249; doi:10.1007/s43441-021-00355-z)
Supplement: Supplementary file 1 — Supplementary file1 (DOCX 39 kb) [file 43441_2021_355_MOESM1_ESM.docx]

**Supplementary material：information of all 36 clusters of AI-based technologies**

The analysis of 140,794 papers resulted in a citation network of 119,553 (85 %) divided into 36 clusters. The number and average year of publications of constituent papers, specific keywords (top10 keywords of TF-ICF, term frequency-inverse cluster frequency, see Methods), and the title of hub-paper of 36 clusters are shown in the Table.

The contents of the top 10 clusters and the clusters deduced from TF-ICF and the title and abstract of hub papers are indicated in the main text. There are several clusters that are considered to be related to the medical field but are not mentioned in the main text. Cluster9 relates to the application of super-resolution to improve the resolution of microscopic images and low-dose CT. It regards the improvement of existing modalities and has a limited range of applications. Cluster 30 that is related to gene expression analysis of biopsy specimens and Cluster 33 relating to the application to the diagnosis of rejection during transplantation are considered to be inappropriate for representing single academic topics, because the number of papers in these clusters is very small, which suggests the possibility of small numbers of researchers’ activities.

| Cluster No. | Average year | The number of papers | Top10 keywords (tf-icf) | Hub paper title |
| --- | --- | --- | --- | --- |
| Cluster1 | 2018.63 | 14033 | convolutional, convolutional neural, convolutional neural network, cnn, remote sensing, image, detection, remote, deep, hyperspectral | ImageNet Classification with Deep Convolutional Neural Networks ^1^ |
| Cluster2 | 2016.37 | 13309 | protein, drug, peptide, gene, ligand, prediction, cancer, sequence, binding, qsar | Random forests ^2^ |
| Cluster3 | 2018.81 | 10992 | segmentation, cancer, radiomics, nodule, lesion, mri, patient, tumor, diagnosis, lung | Deep learning ^3^ |
| Cluster4 | 2014.23 | 9867 | feature selection, ensemble, optimization, fuzzy, selection, classifier, prediction, classification, rule, problem | Statistical comparisons of classifiers over multiple data sets ^4^ |
| Cluster5 | 2017.41 | 7829 | patient, clinical, disease, health, coronary, sepsis, medical, electronic health, care, electronic health record | Predicting the Future - Big Data, Machine Learning, and Clinical Medicine ^5^ |
| Cluster6 | 2018.28 | 7412 | fault, fault diagnosis, diagnosis, deep, convolutional, convolutional neural, rotating machinery, convolutional neural network, speech, cnn | Dropout: A Simple Way to Prevent Neural Networks from Overfitting ^6^ |
| Cluster7 | 2017.98 | 6571 | malware, intrusion detection, attack, intrusion, malware detection, phishing, traffic, detection, intrusion detection system, security | An Introduction to Deep Learning for the Physical Layer ^7^ |
| Cluster8 | 2018.30 | 5815 | forecasting, traffic, energy, load forecasting, traffic, prediction, wind, price, forecast, short term, building | Traffic Flow Prediction With Big Data: A Deep Learning Approach ^8^ |
| Cluster9 | 2018.78 | 4371 | reconstruction, image, super resolution, convolutional, convolutional neural, convolutional neural network, resolution, deep, super, imaging | Image Super-Resolution Using Deep Convolutional Networks ^9^ |
| Cluster10 | 2016.51 | 4333 | sentiment, text, sentiment analysis, social, tweet, word, twitter, social medium, user, sentiment classification | Machine learning in automated text categorization ^10^ |
| Cluster11 | 2018.58 | 4332 | quantum, density functional, material, initio, functional theory, density functional theory, dft, interatomic, alloy, force field | Fast and Accurate Modeling of Molecular Atomization Energies with Machine Learning ^11^ |
| Cluster12 | 2018.25 | 4101 | gait, recognition, activity, action recognition, activity recognition, video, sensor, facial, facial expression, expression recognition | 3D Convolutional Neural Networks for Human Action Recognition ^12^ |
| Cluster13 | 2017.58 | 3800 | disorder, brain, schizophrenia, mci, fmri, mri, suicide, alzheimer, patient, cognitive impairment | Single subject prediction of brain disorders in neuroimaging: Promises and pitfalls ^13^ |
| Cluster14 | 2017.39 | 3705 | landslide, soil, landslide susceptibility, specie distribution, river, flood, water, conditioning factor, climate, habitat | A working guide to boosted regression trees ^14^ |
| Cluster15 | 2018.26 | 3202 | eeg, ecg, seizure, signal, bci, eeg signal, emotion, sleep, ecg signal, arrhythmia | Real-Time Patient-Specific ECG Classification by 1-D Convolutional Neural Networks ^15^ |
| Cluster16 | 2004.54 | 2797 | convolutional code, turbo code, code, turbo, punctured, trellis, ldpc, decoding, punctured convolutional, block code | RATE-COMPATIBLE PUNCTURED CONVOLUTIONAL-CODES (RCPC CODES) AND THEIR APPLICATIONS ^16^ |
| Cluster17 | 2017.87 | 2279 | accelerator, puf, hardware, neuromorphic, crossbar, memory, spiking, spiking neural, memristor, spiking neural network | Eyeriss: An Energy-Efficient Reconfigurable Accelerator for Deep Convolutional Neural Networks ^17^ |
| Cluster18 | 2018.44 | 2234 | crop, plant, weed, fruit, pest, leaf, wheat, agriculture, cow, plant disease | Deep learning in agriculture: A survey ^18^ |
| Cluster19 | 2018.54 | 1893 | seismic, reservoir, earthquake, reynolds stress, shale, reynolds, equation, lithology, physic, flow | Physics-informed neural networks: A deep learning framework for solving forward and inverse problems involving nonlinear partial differential equations ^19^ |
| Cluster20 | 2018.62 | 1724 | crack, pavement, crack detection, defect, damage, damage detection, pavement crack, detection, defect detection, structural | Deep Learning-Based Crack Damage Detection Using Convolutional Neural Networks ^20^ |
| Cluster21 | 2018.00 | 1454 | galaxy, redshift, star, gravitational wave, quasar, pulsar, photometric, photometric redshift, lensing, stellar | Surveying the reach and maturity of machine learning and artificial intelligence in astronomy ^21^ |
| Cluster22 | 2016.27 | 919 | robot, tactile, visual analytics, grasping, tactile sensor, grasp, visual, robotic, tactile sensing, sensor | Deep learning for detecting robotic grasps ^22^ |
| Cluster23 | 2016.27 | 817 | optimization, convex, convergence, stochastic, gradient, kernel, optical music, optical music recognition, svrg, nonconvex | Optimization Methods for Large-Scale Machine Learning ^23^ |
| Cluster24 | 2018.81 | 603 | steganalysis, finger vein, iris, steganography, iris recognition, vein, presentation attack, vein recognition, image steganalysis, finger | Structural Design of Convolutional Neural Networks for Steganalysis ^24^ |
| Cluster25 | 2017.53 | 443 | iqa, image quality, image quality assessment, aesthetic, quality assessment, quality, reference image quality, reference image quality assessment, cognitive component, facial attractiveness | Deep Neural Networks for No-Reference and Full-Reference Image Quality Assessment ^25^ |
| Cluster26 | 2013.04 | 416 | student, surface learning, process questionnaire, study process questionnaire, medical student, education, deep approach, problem based learning, spq, goal orientation | The influence of assessment method on students' learning approaches: Multiple choice question examination versus assignment essay ^26^ |
| Cluster27 | 2013.42 | 136 | cpml, matched layer, pml, fdtd, absorbing boundary, absorbing boundary condition, difference time, difference time domain, finite difference time, finite difference time domain | Convolution PML (CPML): An efficient FDTD implementation of the CFS-PML for arbitrary media ^27^ |
| Cluster28 | 2017.63 | 40 | lithography, hotspot detection, lithography hotspot, opc, optical proximity, proximity correction, optical proximity correction, lithography hotspot detection, hotspot, based opc | Imbalance aware lithography hotspot detection: a deep learning approach ^28^ |
| Cluster29 | 2017.13 | 38 | hrm, nanopores, single molecule, resolution melt, high resolution melt, recognition tunneling, molecule, pcr, aav, tunneling | Trainable High Resolution Melt Curve Machine Learning Classifier for Large-Scale Reliable Genotyping of Sequence Variants ^29^ |
| Cluster30 | 2015.32 | 28 | lymphoma, toxicogenomic, cell lymphoma, opossom, gene, nsnmf, network biomarkers, boolean implication, som portrayal, pathcore | Expression cartography of human tissues using self organizing maps ^30^ |
| Cluster31 | 2018.96 | 24 | cut mark, carnivore, bsm, taphonomy, olduvai, bone surface modification, taphonomic, hominin, olduvai gorge, bone surface | Successful classification of experimental bone surface modifications (BSM) through machine learning algorithms: a solution to the controversial use of BSM in paleoanthropology? ^31^ |
| Cluster32 | 2019.08 | 13 | diffusion index, donnees, donnees massif, term structure, forecasting, une, dynamic regression model, dynamic nelson, dynamic nelson siegel, dns model | Mining big data using parsimonious factor, machine learning, variable selection and shrinkage methods ^32^ |
| Cluster33 | 2019.25 | 8 | tcmr, tbbs, mediated rejection, mucosal biopsy, abmr, biopsy, mucosal, molecular tcmr, transplant biopsy, transplant | Molecular phenotyping of rejection-related changes in mucosal biopsies from lung transplants ^33^ |
| Cluster34 | 2017.67 | 6 | coal, char particle, char, coal characterization, scanned electron, automated coal characterization, scanned electron microscopic, scanned electron microscopic image, coal using image, coal petrology | Machine learning approach for automated coal characterization using scanned electron microscopic images ^34^ |
| Cluster35 | 2010.80 | 5 | shot type, cinematography, svm tree, nonparametric motion, motion pattern descriptor, focal length, natural domain knowledge, loocv protocol, proposed svm tree, shot size | Support-vector-machine tree-based domain knowledge learning toward automated sports video classification ^35^ |
| Cluster36 | 2018.33 | 4 | intensity ranking, yolopeds, tiny pedestrian, simultaneous sensing, intensity ranking image, ranking image sensor, intensity ranking image sensor, intensity ranking image sensor, smart camera, camera application, smart camera application | YOLOpeds: efficient real-time single-shot pedestrian detection for smart camera applications ^36^ |

1. Krizhevsky A, Sutskever I, Hinton GE. ImageNet Classification with Deep Convolutional Neural Networks. *Communications of the Acm* 2017;60(6):84-90. doi: 10.1145/3065386
2. Breiman L. Random forests. *Machine Learning* 2001;45(1):5-32. doi: 10.1023/a:1010933404324
3. LeCun Y, Bengio Y, Hinton G. Deep learning. *nature* 2015;521(7553):436-44.
4. Demsar J. Statistical comparisons of classifiers over multiple data sets. *Journal of Machine Learning Research* 2006;7:1-30.
5. Obermeyer Z, Emanuel EJ. Predicting the Future - Big Data, Machine Learning, and Clinical Medicine. *New England Journal of Medicine* 2016;375(13):1216-19. doi: 10.1056/NEJMp1606181
6. Srivastava N, Hinton G, Krizhevsky A, et al. Dropout: A Simple Way to Prevent Neural Networks from Overfitting. *Journal of Machine Learning Research* 2014;15:1929-58.
7. O'Shea T, Hoydis J. An Introduction to Deep Learning for the Physical Layer. *Ieee Transactions on Cognitive Communications and Networking* 2017;3(4):563-75. doi: 10.1109/tccn.2017.2758370
8. Lv YS, Duan YJ, Kang WW, et al. Traffic Flow Prediction With Big Data: A Deep Learning Approach. *Ieee Transactions on Intelligent Transportation Systems* 2015;16(2):865-73. doi: 10.1109/tits.2014.2345663
9. Dong C, Loy CC, He KM, et al. Image Super-Resolution Using Deep Convolutional Networks. *Ieee Transactions on Pattern Analysis and Machine Intelligence* 2016;38(2):295-307. doi: 10.1109/tpami.2015.2439281
10. Sebastiani F. Machine learning in automated text categorization. *ACM computing surveys (CSUR)* 2002;34(1):1-47.
11. Rupp M, Tkatchenko A, Muller KR, et al. Fast and Accurate Modeling of Molecular Atomization Energies with Machine Learning. *Physical Review Letters* 2012;108(5) doi: 10.1103/PhysRevLett.108.058301
12. Ji SW, Xu W, Yang M, et al. 3D Convolutional Neural Networks for Human Action Recognition. *Ieee Transactions on Pattern Analysis and Machine Intelligence* 2013;35(1):221-31. doi: 10.1109/tpami.2012.59
13. Arbabshirani MR, Plis S, Sui J, et al. Single subject prediction of brain disorders in neuroimaging: Promises and pitfalls. *Neuroimage* 2017;145:137-65. doi: 10.1016/j.neuroimage.2016.02.079
14. Elith J, Leathwick JR, Hastie T. A working guide to boosted regression trees. *Journal of Animal Ecology* 2008;77(4):802-13. doi: 10.1111/j.1365-2656.2008.01390.x
15. Kiranyaz S, Ince T, Gabbouj M. Real-Time Patient-Specific ECG Classification by 1-D Convolutional Neural Networks. *Ieee Transactions on Biomedical Engineering* 2016;63(3):664-75. doi: 10.1109/tbme.2015.2468589
16. Hagenauer J. RATE-COMPATIBLE PUNCTURED CONVOLUTIONAL-CODES (RCPC CODES) AND THEIR APPLICATIONS. *Ieee Transactions on Communications* 1988;36(4):389-400. doi: 10.1109/26.2763
17. Chen YH, Krishna T, Emer JS, et al. Eyeriss: An Energy-Efficient Reconfigurable Accelerator for Deep Convolutional Neural Networks. *Ieee Journal of Solid-State Circuits* 2017;52(1):127-38. doi: 10.1109/jssc.2016.2616357
18. Kamilaris A, Prenafeta-Boldu FX. Deep learning in agriculture: A survey. *Computers and Electronics in Agriculture* 2018;147:70-90. doi: 10.1016/j.compag.2018.02.016
19. Raissi M, Perdikaris P, Karniadakis GE. Physics-informed neural networks: A deep learning framework for solving forward and inverse problems involving nonlinear partial differential equations. *Journal of Computational Physics* 2019;378:686-707. doi: 10.1016/j.jcp.2018.10.045
20. Cha YJ, Choi W, Buyukozturk O. Deep Learning-Based Crack Damage Detection Using Convolutional Neural Networks. *Computer-Aided Civil and Infrastructure Engineering* 2017;32(5):361-78. doi: 10.1111/mice.12263
21. Fluke CJ, Jacobs C. Surveying the reach and maturity of machine learning and artificial intelligence in astronomy. *Wiley Interdisciplinary Reviews-Data Mining and Knowledge Discovery* 2020;10(2) doi: 10.1002/widm.1349
22. Lenz I, Lee H, Saxena A. Deep learning for detecting robotic grasps. *International Journal of Robotics Research* 2015;34(4-5):705-24. doi: 10.1177/0278364914549607
23. Bottou L, Curtis FE, Nocedal J. Optimization Methods for Large-Scale Machine Learning. *Siam Review* 2018;60(2):223-311. doi: 10.1137/16m1080173
24. Xu GS, Wu HZ, Shi YQ. Structural Design of Convolutional Neural Networks for Steganalysis. *Ieee Signal Processing Letters* 2016;23(5):708-12. doi: 10.1109/lsp.2016.2548421
25. Bosse S, Maniry D, Muller KR, et al. Deep Neural Networks for No-Reference and Full-Reference Image Quality Assessment. *Ieee Transactions on Image Processing* 2018;27(1):206-19. doi: 10.1109/tip.2017.2760518
26. Scouller K. The influence of assessment method on students' learning approaches: Multiple choice question examination versus assignment essay. *Higher Education* 1998;35(4):453-72. doi: 10.1023/a:1003196224280
27. Roden JA, Gedney SD. Convolution PML (CPML): An efficient FDTD implementation of the CFS-PML for arbitrary media. *Microwave and Optical Technology Letters* 2000;27(5):334-39. doi: 10.1002/1098-2760(20001205)27:5<334::aid-mop14>3.3.co;2-1
28. Yang HY, Luo LY, Su J, et al. Imbalance aware lithography hotspot detection: a deep learning approach. *Journal of Micro-Nanolithography Mems and Moems* 2017;16(3) doi: 10.1117/1.jmm.16.3.033504
29. Athamanolap P, Parekh V, Fraley SI, et al. Trainable High Resolution Melt Curve Machine Learning Classifier for Large-Scale Reliable Genotyping of Sequence Variants. *Plos One* 2014;9(10) doi: 10.1371/journal.pone.0109094
30. Wirth H, Loffler M, von Bergen M, et al. Expression cartography of human tissues using self organizing maps. *Bmc Bioinformatics* 2011;12 doi: 10.1186/1471-2105-12-306
31. Dominguez-Rodrigo M. Successful classification of experimental bone surface modifications (BSM) through machine learning algorithms: a solution to the controversial use of BSM in paleoanthropology? *Archaeological and Anthropological Sciences* 2019;11(6):2711-25. doi: 10.1007/s12520-018-0684-9
32. Kim HH, Swanson NR. Mining big data using parsimonious factor, machine learning, variable selection and shrinkage methods. *International Journal of Forecasting* 2018;34(2):339-54. doi: 10.1016/j.ijforecast.2016.02.012
33. Halloran K, Parkes MD, Timofte IL, et al. Molecular phenotyping of rejection-related changes in mucosal biopsies from lung transplants. *American Journal of Transplantation* 2020;20(4):954-66. doi: 10.1111/ajt.15685
34. Alpana, Mohapatra S. Machine learning approach for automated coal characterization using scanned electron microscopic images. *Computers in Industry* 2016;75:35-45. doi: 10.1016/j.compind.2015.10.003
35. Xiao GQ, Jiang Y, Song G, et al. Support-vector-machine tree-based domain knowledge learning toward automated sports video classification. *Optical Engineering* 2010;49(12) doi: 10.1117/1.3518080
36. Kyrkou C. YOLOpeds: efficient real-time single-shot pedestrian detection for smart camera applications. *Iet Computer Vision* 2020;14(7):417-25. doi: 10.1049/iet-cvi.2019.0897
